# PEGylation of mRNA by Hybridization of Complementary PEG-RNA Oligonucleotides Stabilizes mRNA without Using Cationic Materials

**DOI:** 10.3390/pharmaceutics13060800

**Published:** 2021-05-27

**Authors:** Naoto Yoshinaga, Mitsuru Naito, Yoshihiro Tachihara, Eger Boonstra, Kensuke Osada, Horacio Cabral, Satoshi Uchida

**Affiliations:** 1 Graduate School of Engineering, The University of Tokyo, 7-3-1 Hongo, Bunkyo-ku, Tokyo 113-8656, Japan; naoto.yoshinaga@riken.jp (N.Y.); tachihara@g.ecc.u-tokyo.ac.jp (Y.T.); eger@g.ecc.u-tokyo.ac.jp (E.B.); 2 RIKEN Center for Sustainable Resource Science, Wako 351-0198, Saitama, Japan; 3 Graduate School of Medicine, The University of Tokyo, 7-3-1 Hongo, Bunkyo-ku, Tokyo 113-0033, Japan; naito@bmw.t.u-tokyo.ac.jp; 4 National Institute of Radiological Science, 4-9-1 Anagawa, Inage-ku, Chiba-shi 236-8555, Chiba, Japan; osada.kensuke@qst.go.jp; 5 Graduate School of Medical Science, Kyoto Prefectural University of Medicine, Inamori Memorial Building, 1-5 Shimogamohangi-cho, Sakyo-ku, Kyoto 606-0823, Japan; 6 Innovation Center of NanoMedicine (iCONM), Kawasaki Institute of Industrial Promotion, 3-25-14 Tonomachi, Kawasaki-ku, Kawasaki 210-0821, Japan

**Keywords:** mRNA therapeutics, RNA engineering, mRNA delivery

## Abstract

Messenger RNA (mRNA) delivery strategies are required to protect biologically fragile mRNA from ribonuclease (RNase) attacks to achieve efficient therapeutic protein expression. To tackle this issue, most mRNA delivery systems have used cationic components, which form electrostatically driven complexes with mRNA and shield encapsulated mRNA strands. However, cationic materials interact with anionic biomacromolecules in physiological environments, which leads to unspecific reactions and toxicities. To circumvent this issue of cation-based approaches, herein, we propose a cation-free delivery strategy by hybridization of PEGylated RNA oligonucleotides with mRNA. The PEG strands on the mRNA sterically and electrostatically shielded the mRNA, improving mRNA nuclease stability 15-fold after serum incubation compared with unhybridized mRNA. Eventually, the PEGylated mRNA induced nearly 20-fold higher efficiency of reporter protein expression than unhybridized mRNA in cultured cells. This study provides a platform to establish a safe and efficient cation-free mRNA delivery system.

## 1. Introduction

In recent years, messenger RNA (mRNA) has been widely used for medical applications, supplying therapeutic proteins at proper timing in a safe manner [1,2,3]. Nevertheless, the biological fragility of mRNA against ribonuclease (RNase) attacks impedes the development of mRNA applications for in vivo use. In addition, exogenous mRNA strands are recognized by pattern recognition receptors (PRRs) [4,5], such as Toll-like receptor and retinoic acid-inducible gene I (RIG-I), causing a strong immune response. To solve this problem, mRNA is usually complexed with cationic lipids or polymers to avoid enzymatic degradation and PRR recognition en route to the target sites [6,7,8,9]. Cationic materials can also be designed to modulate the intracellular processes, for example, by facilitating endosomal escape [10] and preventing intracellular mRNA degradation [11]. Furthermore, pharmacological functionalities, such as adjuvanticity in vaccination, can be integrated to the cationic materials [12,13]. The complexation strategy exhibits potent outcomes in efficient mRNA delivery. However, as cationic materials interact with anionic biomacromolecules, such as glycosaminoglycans on the cell surface [14], cationic carrier components frequently damage cells and tissues [15,16,17]. Furthermore, complexed mRNA can be kicked out by the exchange reaction with anionic biomacromolecules, leading to enzymatic degradation of mRNA. These issues derived from cationic materials have accelerated the development of cation-free delivery strategies in the field of mRNA applications [18], and, in fact, some clinical trials have used naked mRNA in spite of the biological fragility and immunogenicity of naked mRNA [19,20]. The increased momentum of cation-free mRNA delivery strategies motivated us to design a safe and efficient system without using cationic materials.

**Figure 1:**
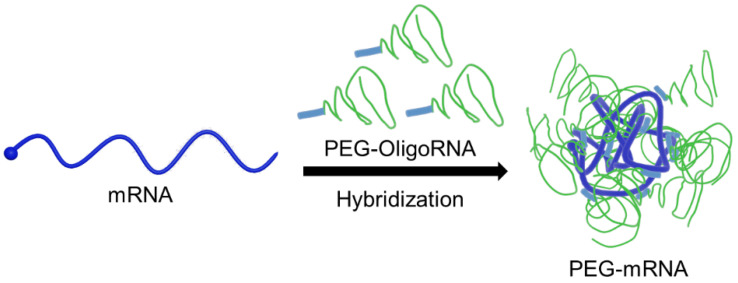
Schematic illustration of PEG-mRNA. mRNA is directly PEGylated through hybridization of PEG-OligoRNA, and the PEG strands protect mRNA from enzymatic degradation.

Chemical modification of nucleotides in mRNA is an effective method to improve the bioavailability of mRNA, especially to reduce the immunogenicity of mRNA [21,22,23]. However, currently used chemical modifications provide minimal protection of mRNA against RNases under physiological conditions [24]. Thus, additional methods are needed to modify mRNA without using cationic materials. Recently, we reported an innovative technology to functionalize mRNA through hybridization with complementary RNA oligonucleotides (OligoRNAs) equipped with a desired moiety [25,26,27]. Fine-tuning of the complementary length of OligoRNA sequences, specifically 17 nt of complementary length, enables mRNA modification without decreasing mRNA translational activity and without inducing an immune response caused by double strand RNA (dsRNA) regions. Ultimately, we demonstrated the utility of this approach to improve the functionalities of polycation-based mRNA delivery systems. In this study, we employed this method to improve RNase resistance without using cationic materials by directly PEGylating mRNA (PEG-mRNA) using 17 nt OligoRNAs possessing a PEG strand (PEG-OligoRNA) (Figure 1). The PEG strands surrounding the mRNA were expected to reduce non-specific interaction with charged macromolecules in physiological conditions, leading the inhibition of unpreferable recognition by RNases for efficient mRNA delivery. Indeed, PEG-OligoRNA hybridization with mRNA enhanced the tolerability against RNases and eventually improved the introduction efficiency of PEG-mRNA in cultured cells. For future clinical application, it is important to note that our system is composed only of mRNA and PEG, which has already been approved for clinical usage in many drugs. On the other hand, clinical approval of new materials requires laborious processes, except when a pandemic situation prompts approval for emergency usage.

## 2. Materials and Methods

### 2.1. Preparation of PEG-mRNA through Hybridization of PEG-OligoRNA

mRNA encoding *Gaussia luciferase* (*GLuc*) with a 120 base poly A sequence was in vitro transcribed according to a previous report [28]. The concentration of the prepared mRNA was determined using a NanoDrop 1000 spectrophotometer (NanoDrop Technologies Inc., Wilmington, DE, USA). In addition, successful mRNA preparation was checked by on-chip capillary electrophoresis using Bioanalyzer Agilent2100 (Agilent, Santa Clare, CA, USA) at 25 ng/µL of mRNA.

mRNA was then PEGylated through hybridization of PEG-OligoRNAs complementary to the target sequences on *GLuc* mRNA (GeneDesign Inc., Osaka, Japan), which are composed of 17 nt RNA oligonucleotides with 12 kDa PEG at the 5′ end according to a previous publication [26]. The molar ratio of each sequence of PEG-OligoRNA to mRNA was controlled to be 1:1. The PEG-OligoRNA sequences are described in Supplementary Table S1. mRNA hybridized with PEG-OligoRNAs (mRNA concentration: 100 ng/µL) was evaluated by dynamic light scattering (DLS) measurement with three repeats at 25 °C in 10 mM HEPES buffer using a Zetasizer Nano (Zetasizer Nano-ZS, Malvern Instruments, Worcestershire, UK.) equipped with a 532 nm laser.

### 2.2. Potential Measurement of PEG-mRNA

The ζ-potential of PEG-mRNA was measured at 25 °C in 10 mM HEPES buffer by laser-doppler electrophoresis using Möbius ζ™ (Wyatt Technology Corporation, Santa Barbara, CA, USA) with a 532 nm laser according to the calculation based on the Smoluchowski equation. ζ-potential measurements were conducted three times for each sample, and the average of all 3 measurements is presented.

### 2.3. Atomic Force Microscopy (AFM) Observation

MgCl_2_ solution (10 mM) was added onto MICA, followed by the addition of PEG-mRNA solution with a final mRNA concentration of 5.55 ng/μL. After a 1 min incubation, MICA was washed once with distilled water. AFM observations were conducted in the tapping mode under the air phase mode using SPM9700 (Shimadzu Co., Kyoto, Japan) with a micro cantilever OMCL-AC240TS-C2 (70 kHz resonance frequency and 2 N/m spring constant; Olympus Co., Tokyo, Japan). Obtained AFM images were processed by flattening them to remove the background using software. Height, long axis, and short axis were calculated by measuring 100 individual particles.

### 2.4. Nuclease Resistance of PEG-mRNA in FBS Solution

The PEG-mRNA solutions at 16.7 µg/mL of mRNA were incubated for 15 min at 37 °C in 24 µL of 10 mM HEPES buffer with 150 mM NaCl and 1 *v*/*v*% FBS. To assess the effect of PEG strands, 0*PEG-mRNA (16.7 µg/mL) with PEG (11.3 µg/mL), which was the same concentration in 15*PEG-mRNA, was also incubated in the same condition. mRNA samples incubated in 10 mM HEPES buffer containing 150 mM NaCl without FBS were used as a control for each group. After incubation, 350 µL of RLT buffer from an RNeasy Mini kit (Qiagen, Hilden, Germany) containing 1% 2-mercaptoethanol was added to the samples. The mixtures were denatured by incubation at 65 °C for 5 min, followed by being put on ice immediately. mRNA was then extracted from the denatured samples using an RNeasy Mini kit according to the manufacturer’s protocol. After reverse transcription of extracted mRNA using a ReverTra Ace qPCR RT Master Mix kit, quantitative real-time PCR (qRT-PCR) analysis was conducted using an ABI Prism 7500 Sequence Detector and primer pairs (forward: TGAGATTCCTGGGTTCAAGG; reverse: GTCAGAACACTGCACGTTGG).

### 2.5. Transfection of PEG-mRNA into Cultured Cells

Human hepatoma cell line (HuH-7) obtained from RIKEN cell bank (Tsukuba, Japan) was seeded on 96-well plate at a density of 10,000 cells/well in Dulbecco’s modified Eagle’s medium (DMEM) (Sigma-Aldrich, St. Louis, MO, USA) containing 10% FBS (Dainippon Sumitomo Pharma Co., Ltd., Osaka, Japan) and 1% penicillin/streptomycin (Sigma-Aldrich, St. Louis, MO, USA) in a humidified atmosphere with 5% CO_2_ at 37 °C. After 24 h incubation, the culture medium was replaced with 100 µL of Opti-MEM (Thermo Fisher Scientific, Waltham, MA, USA), followed by adding 7.5 µL of PEG-mRNA containing 250 ng of mRNA. After 4 h, 10 µL of cell culture medium was assessed using a Renilla luciferase assay kit (Promega Co., Madison, WI, USA) and a luminometer (Mithras LB940, Berthed Technologies, Bad Wildbad, Germany).

## 3. Results

### 3.1. Characterization of PEG-mRNA

**Figure 2:**
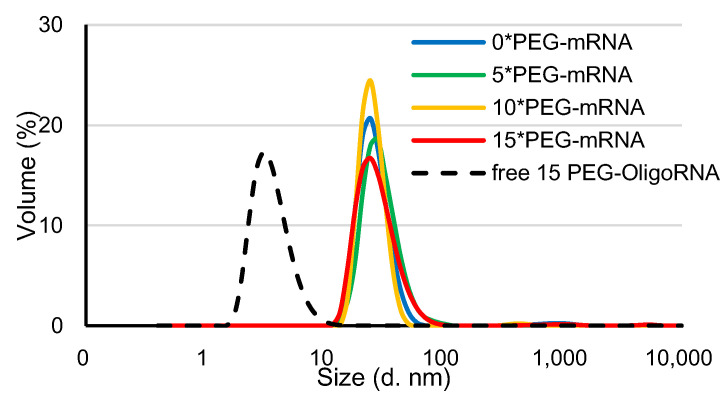
DLS measurement of n*PEG-mRNA at 25 °C. Solid and dotted line represent n*PEG-mRNA samples and PEG-OligoRNA mixture, respectively.

PEG-mRNA was prepared by hybridization of 5′ end-PEGylated OligoRNA (PEG-OligoRNA), with the molar ratio of each sequence of PEG-OligoRNA to mRNA of 1:1, according to a previous publication [26]. In this study, 15 sequences of PEG-OligoRNA were designed for mRNA encoding *Gaussia luciferase* (*GLuc*: 783 nt) at different regions, except the start codon and poly A tail, which are known to be critical regions for mRNA translation (Supplementary Figure S1). Moreover, in the PEG-OligoRNA design, we avoided dsRNA regions predicted by IPKnot software (http://rtips.dna.bio.keio.ac.jp/ipknot/, accessed on 15 January 2018) [29], because such dsRNA regions may compete with PEG-OligoRNA and prevent efficient hybridization. In the following experiment, mRNA was hybridized with 5, 10, or 15 PEG-OligoRNAs, denoted as n*PEG-mRNA (*n* = 5, 10 or 15). Additionally, the mRNA without PEG-OligoRNA hybridization was denoted as 0*PEG-mRNA. According to gel permeation chromatography (GPC), almost all PEG-OligoRNA was successfully hybridized to mRNA, as was the case in our previous report (Supplementary Figure S2 and Table S2) [26]. The prepared n*PEG-mRNA was analyzed using DLS measurements. The cumulant diameter of n*PEG-mRNA was in the range of 10–90 nm, while the free PEG-OligoRNA mixture containing 15 different sequences was 2–10 nm (Figure 2). The shielding effect of PEG strands on PEG-mRNA was then assessed through ζ-potential measurement. The ζ-potential value of n*PEG-mRNA became closer to neutral with an increasing number of PEG strands on mRNA (Table 1), indicating the successful shielding of mRNA by PEG-OligoRNAs.

### 3.2. AFM Observation of PEG-mRNA

**Figure 3:**
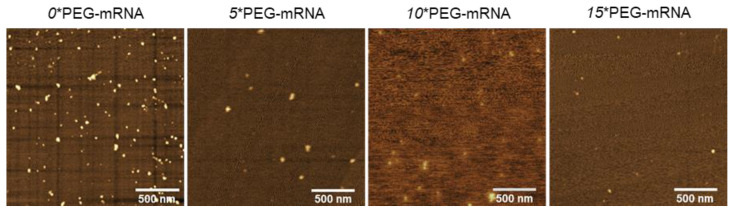
Representative images of AFM observation of n*PEG-mRNA.

The structure of n*PEG-mRNA was observed by AFM (Figure 3). The number of observed particles in n*PEG-mRNA samples (*n* = 5, 10, and 15) was less than that in 0*PEG-mRNA, presumably because PEG chains may hamper the attachment of mRNA to MICA surfaces. As poorly structured polymer strands are undetectable by AFM observation [30], it is reasonable to assume that the particles observed in Figure 3 represent mRNA strands, with the remaining PEG chain being invisible. Intriguingly, the size of the mRNA remained small after increasing the number of PEG-OligoRNAs (Table 2) without obvious changes in structure, despite the formation of rigid dsRNA structures in the PEG-OligoRNAs, which potentially stretch mRNA strands. This observation suggests that PEG chains surrounding the mRNA become denser after the increase in PEG-OligoRNA numbers.

### 3.3. Nuclease Resistance of PEG-mRNA

**Figure 4:**
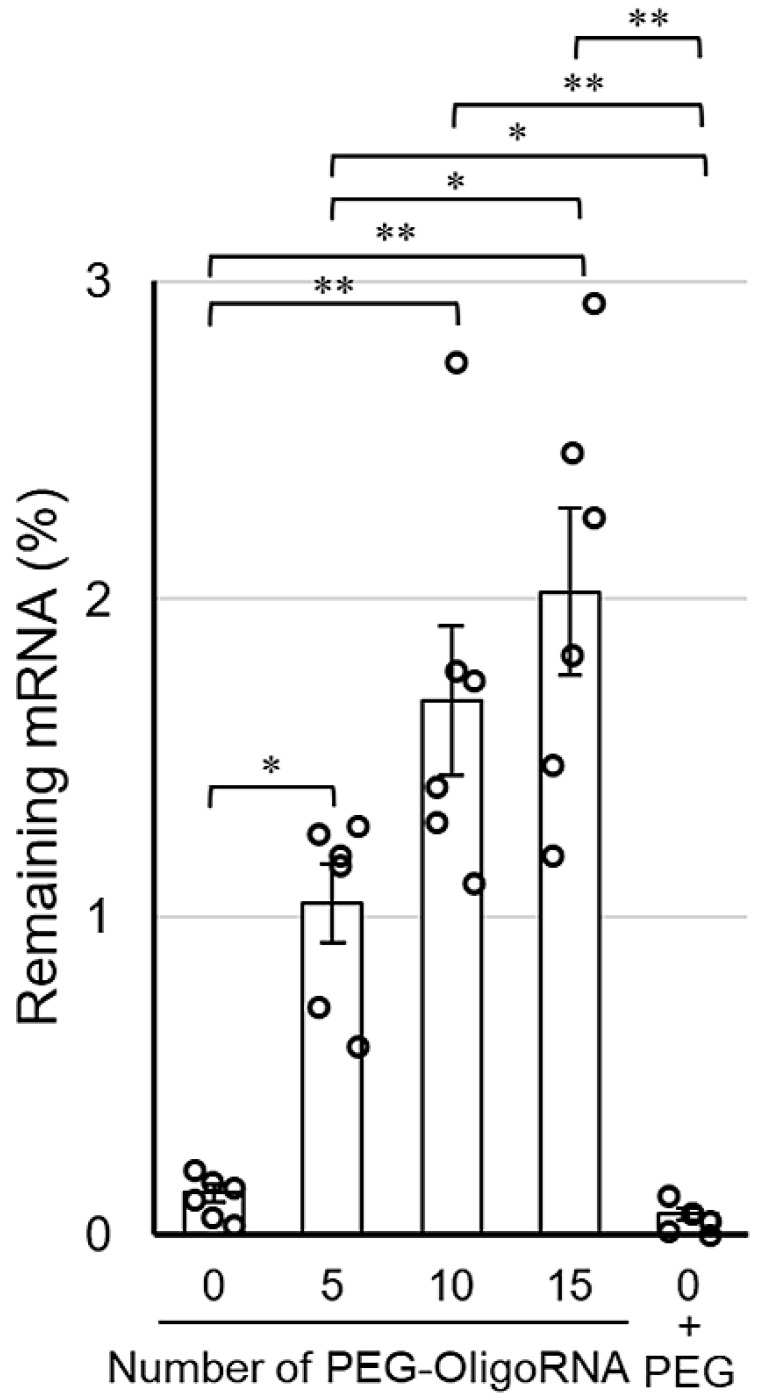
Remaining amount of mRNA after incubation in 1% FBS solution for 15 min. Data are shown as average mean ± SEM. Each dot refers to an individual measurement (*n* = 6). Statistical difference was analyzed by ANOVA followed by Tukey’s test. Abbr., 0 + PEG: 0*PEG-mRNA in the presence of free PEG strands. * *p* < 0.05, ** *p* < 0.01.

The stabilization by hybridization of PEG-OligoRNA against RNases was evaluated by quantifying intact mRNA using qRT-PCR after incubation in FBS solution, which gives an experimental condition relevant to an in vivo environment. Notably, as RNases in FBS are a dominant factor in degrading mRNA [25], this method accurately reflects the tolerability of mRNA against RNases. After 15 min incubation in 1% FBS solution, the remaining mRNA amount in n*PEG-mRNA (*n* = 10, and 15) was approximately 10–15-fold higher than that of 0*PEG-mRNA (Figure 4). Additionally, 0*PEG-mRNA mixed with free PEG strands failed to improve nuclease resistance. As the hybridization of unmodified OligoRNA does not enhance mRNA tolerability against nucleases [31], these results indicate the PEG strands on OligoRNA protect mRNA from nuclease degradation. Interestingly, 15*PEG-mRNA exhibited a gradual decrease in remaining mRNA amount by prolonging incubation time from 5 to 15 min (Supplementary Figure S3). These results indicate that the PEG strands surrounding the mRNA prevented RNases from accessing the mRNA strand, which slowed down the enzymatic degradation processes.

### 3.4. Transfection of PEG-mRNA in Cultured Cells

**Figure 5:**
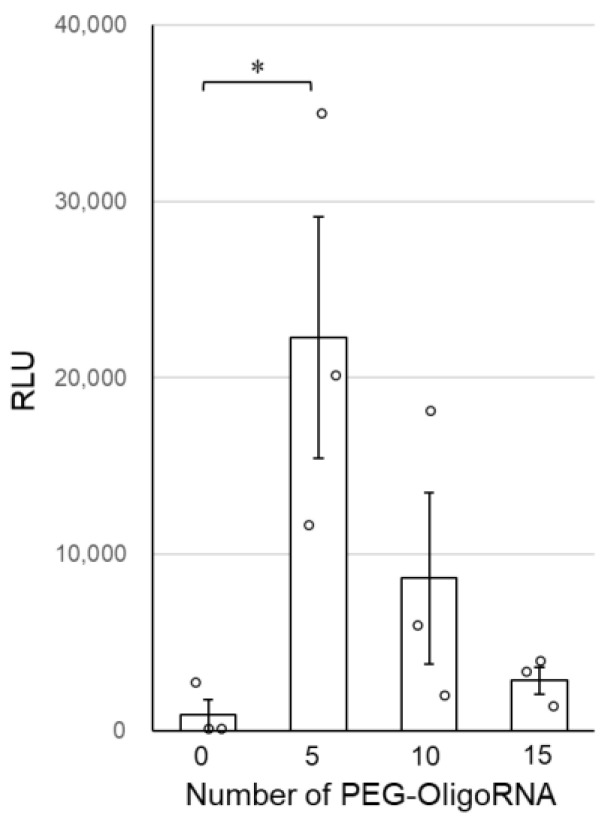
Transfection efficiency of n*PEG-mRNA in cultured cells. Data are shown as average mean ± SEM. Each dot refers to an individual measurement (*n* = 3). Statistical difference was analyzed by ANOVA followed by Dunnett’s test as compared with the 0*PEG-mRNA. * *p* < 0.05.

Finally, the transfection activity of n*PEG-mRNA was investigated by quantifying GLuc expression in cultured cells to study the relation between nuclease stability and mRNA expression efficiency. GLuc expression efficiency tended to improve after PEG-OligoRNA hybridization, with 5*PEG-mRNA showing a nearly 20-fold increase in GLuc expression levels compared to 0*PEG-mRNA (Figure 5). Note that cell viability was almost 100% in all groups 24 h after the transfection (Supplementary Figure S4).

## 4. Discussion

The rapid progress in mRNA delivery technologies using cationic materials has made mRNA applicable to therapeutics, but cationic components may restrict the widespread usage of mRNA due to the non-negligible cytotoxicity. Traditionally, physical delivery methods, such as gene guns and electroporation, were used as cation-free transfection strategies [32,33]. However, as these methods can excessively damage both target and non-target cells [34], these issues also restrict delivery systems based on physical approaches for practical application. In this study, we proposed a simple yet effective cation-free strategy by directly PEGylating mRNA via the hybridization of complementary OligoRNA. The PEG-OligoRNA hybridization strategy dramatically improved the tolerability of mRNA against enzymatic degradation without using cationic materials (Figure 4), leading to nearly 20-fold higher translational activity of mRNA in the cultured cells compared with unhybridized mRNA (Figure 5). As the dsRNA formation by hybridization provided only a modest protection effect on mRNA against RNases [31], the drastic improvement of RNase resistance by PEG-OligoRNA hybridization may be attributed to other mechanisms, especially PEG coating inhibiting RNase recognition of mRNA. This technology is promising for practical mRNA delivery, without the use of toxic cationic materials. Notably, our strategy of PEG-OligoRNA hybridization may be applicable to other mRNA encoding different proteins, regardless of the length of mRNA, as the stabilization effect should be dependent on PEG density on mRNA. Furthermore, while mRNA complexed with cationic components can be replaced through polyion exchange reactions with anionic biomacromolecules and be immediately degraded by RNases [27,35], the current strategy of directly PEGylating mRNA is not affected by the exchange events.

In AFM observations, the size of the mRNA remained small after increasing the number of PEG-OligoRNA, without obvious changes in structure, suggesting that PEG chains surrounding the mRNA became denser after the increase in PEG-OligoRNA numbers. In accordance with this observation, hybridization of a larger number of PEG-OligoRNAs led to enhanced resistance of mRNA against RNases. To gain further insight into this issue, we estimated the volume of the surrounding space of mRNA occupied by PEG strands. The hydrodynamic diameter (R_H_) of one PEG chain with a M_w_ of 12 kDa in an aqueous solution is theoretically calculated to be 3.2 nm from the radius of gyration of the PEG chain [36,37]. In the case of 15*PEG-mRNA, 15 PEG strands occupied 1.9 × 10^3^ nm^3^ (=14 × 4π(3.2)^3^/3 nm^3^) per mRNA strand, and mRNA, which is regarded as an ellipsoid, occupied 7.8 × 10 nm^3^ (=4π(0.9/2)(16.5/2)(10.0/2)/3 nm^3^) in AFM images. Thus, the mRNA and PEG are estimated to occupy 2.0 × 10^3^ nm^3^ in total. Meanwhile, as the R_H_ of 15*PEG-mRNA was approximately 12 nm in DLS measurements (Figure 2), 15*PEG-mRNA was calculated to occupy 7.2 × 10^3^ nm^3^ (=4π(12)^3^/3 nm^3^), which was larger than the total space occupied by PEG and mRNA (2.0 × 10^3^ nm^3^). This indicates that there are still regions unoccupied by PEG chains even after 15 PEG-OligoRNA hybridization. Notably, even such modest levels of PEG coating drastically improved nuclease stability of mRNA (Figure 4).

Although the tolerability of mRNA against RNases was improved by increasing the number of hybridized PEG-OligoRNAs, mRNA introduction efficiency in cultured cells became maximal in 5*PEG-mRNA, with the efficiency becoming lower in 10*PEG-mRNA and 15*PEG-mRNA (Figure 5). Excess PEGylation of mRNA might inhibit the interaction of PEG-mRNA with cell and/or endosomal membranes, hampering its translocation to the cytoplasm. According to previous mechanistic studies, naked mRNA is first recognized by scavenger receptors, which mediate cell entry of negatively charged macromolecules [38,39], followed by internalization through caveolae and/or a lipid rafts-dependent endocytic pathway [40,41,42]. This pathway may be disturbed after PEGylation of mRNA, driving ζ-potential of the mRNA closer to neutral (Table 1). To solve such a dilemma of PEG shielding, many efforts have been devoted to developing effective delivery strategies, such as the introduction of cleavable linkers to release PEG strands in response to a specific environment [43] and the installation of ligand molecules at the end of the PEG strand enhancing cellular uptake [9,44]. Moreover, the PEG strand can be replaced with alternatives, such as zwitterionic polymers [45]. As the PEG-mRNA system can be knitted together with these methodologies without any conflict, we plan to integrate the strategies overcoming the PEG dilemma into PEG-mRNA for more efficient mRNA delivery.

In conclusion, we succeeded at improving the nuclease stability of mRNA without the use of cationic materials, by directly PEGylating mRNA using complementary PEG-OligoRNAs. This simple yet effective strategy is promising for circumventing the annoying toxic effects of cationic materials, which motivates us to plan in vivo experiments to sublimate PEG-mRNA for therapeutic applications in future studies. Meanwhile, the nuclease stability of PEG-mRNA is still lower than that of polycation-based systems. For example, polyplex micelles from PEG-PAsp(DET) block copolymer that showed therapeutic outcomes in animal models of various diseases [9] exhibited approximately 6% of remaining mRNA after incubation for 15 min in a 10% FBS condition [25]. While a further increase in the number of PEG-OligoRNAs might modestly increase the nuclease stability of mRNA, the increase may also result in the decrease in transfection efficiency as shown in Figure 5. Thus, combination with other cation-free approaches is needed to improve nuclease stability and transfection efficiency of mRNA. The approaches include mRNA bundling [31], decationization of carrier components [46], and reduction of RNase recognition by phosphorothioate modification of mRNA [47]. The approach of mRNA bundling improves the nuclease stability of mRNA to the extent comparable with PEG-OligoRNA hybridization. While the decationization strategy has a potential to improve mRNA nuclease stability more effectively than PEG-OligoRNA hybridization, this approach requires additional materials for stabilization. In contrast, our system is composed only of mRNA and PEG, which have already been approved for clinical usage in many drugs. Phosphorothioate modification might have a toxicity concern despite its promises as demonstrated in oligonucleotide therapeutics. While each approach has its own pros and cons, synergistic effects are expected by the combination with PEG-OligoRNA hybridization.

## Figures and Tables

**Table 1:** ζ-potential measurement of n*PEG-mRNA at 25 °C. Data are shown as average mean ± SEM (*n* = 3).

**Number of PEG-OligoRNA**	0	5	10	15
**ζ-potential (mV)**	−31.2 ± 5.0	−24.6 ± 3.7	−25.0 ± 4.5	−16.4 ± 0.8

**Table 2:** Characterization of n*PEG-mRNA from AFM images. The shape of n*PEG-mRNA was assumed to be an ellipsoid. Data are shown as average mean ± SD calculated from 100 individual particles.

**Number of PEG-OligoRNA**	0	5	10	15
**Height (nm)**	3.2 ± 1.2	1.0 ± 0.4	1.1 ± 0.5	0.9 ± 0.5
**Long Axis (nm)**	36.5 ± 14.9	24.2 ± 7.7	20.4 ± 10.6	16.5 ± 8.9
**Short Axis (nm)**	26.4 ± 8.8	16.5 ± 5.4	13.1 ± 8.2	10.0 ± 5.1

## Supplementary Materials

The following are available online at https://www.mdpi.com/article/10.3390/pharmaceutics13060800/s1, Figure S1: Schematic illustration of the PEG-OligoRNA location on *GLuc* mRNA, Figure S2: Gel permeation chromatography to evaluate hybridization efficiency of PEG-OligoRNA, Figure S3: Time dependence of remaining amount of mRNA after incubation in FBS solution, Figure S4: Cell viability after treatment of *n**PEG-mRNA, Table S1: Sequences of PEG-OligoRNAs, and Table S2: The number of hybridized PEG-OliogRNA with mRNA.
