# Supplementary material for: PEGylation of mRNA by Hybridization of Complementary PEG-RNA Oligonucleotides Stabilizes mRNA without Using Cationic Materials
Source: Pharmaceutics. 2021 May 27;13(6):800. doi: 10.3390/pharmaceutics13060800 (PMC8227728; doi:10.3390/pharmaceutics13060800)
Supplement: Supplementary file 1 [file pharmaceutics-13-00800-s001.zip › pharmaceutics-1198525-supplementary.pdf]

pharmaceutics

MDPI

Supplementary Materials: PEGylation of mRNA by Hybridization of Complementary PEG-RNA Oligonucleotides Stabilizes mRNA without Using Cationic Materials

Naoto Yoshinaga, Mitsuru Naito, Yoshihiro Tachihara, Eger Boonstra, Kensuke Osada, Horacio Cabral and Satoshi Uchida

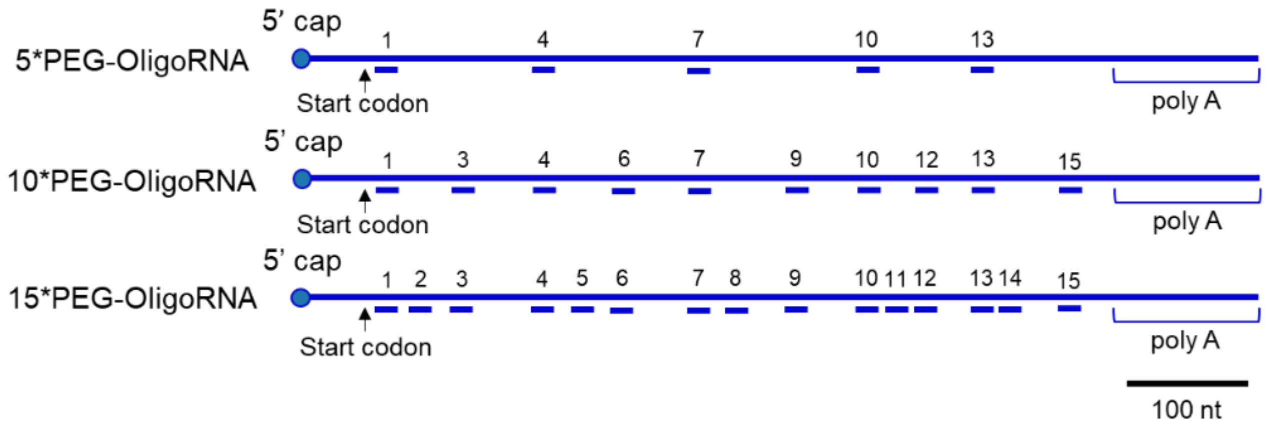

Figure S1. Schematic illustration of the location of PEG-OligoRNA on GLucmRNA.

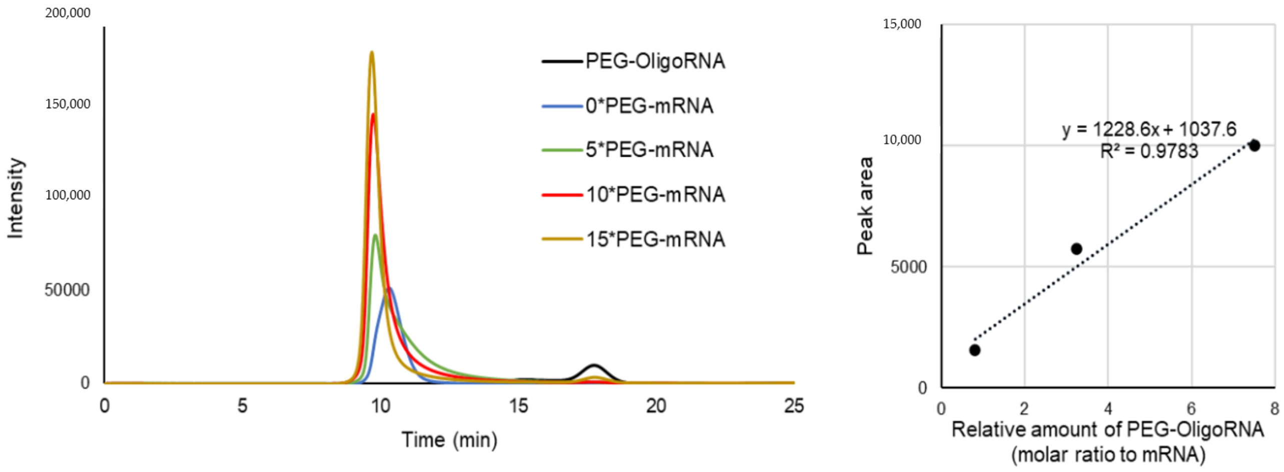

Figure S2. Gel permeation chromatography (GPC) performed using JASCO UV-4075 (JASCO, Tokyo, Japan) equipped with Sephadex 200 Increase 10/300 GL column (GE Healthcare UK Ltd., Buckinghamshire, UK) in 10 mM HEPES buffer containing 150 mM NaCl at flow rate of 0.75 ml/min.(left) GPC chart of *n**PEG-mRNA and free PEG-OligoRNA solutions.The peaks in 9–12 min are derived from *n**PEG-mRNA, and those in 17–19 min are from free PEG-OligoRNA.(right) Standard curve prepared from peak area of free PEG-OligoRNA solutions with different concentration. The amount of free PEG-OligoRNA in the *n**PEG-mRNA solution was calculated based on the peak area from free PEG-OligoRNA in GPC chartand standard curve.

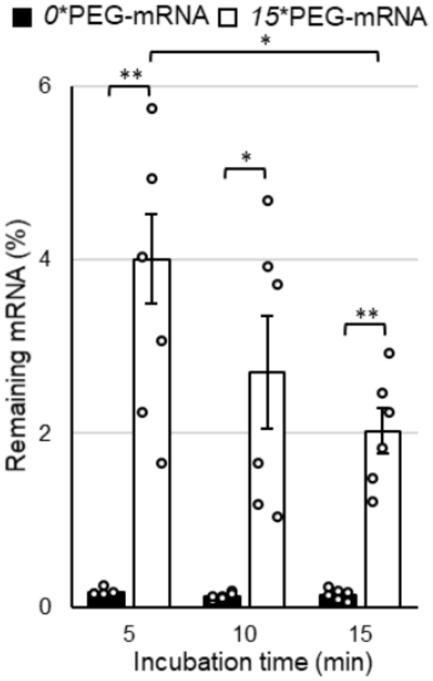

Figure S3. Remaining amount of mRNA in 0*PEG-mRNA and 15*PEG-mRNA after incubation in 1% FBS solution. Data are shown as average mean ± SEM. Each dot refers to an individual measurement ($n = 6$). The data after15 min incubation is thesame as that in Figure 4. Statistical difference was analyzed by 2-tailed unpaired Student's t test. * $p < 0.05$, ** $p < 0.01$.

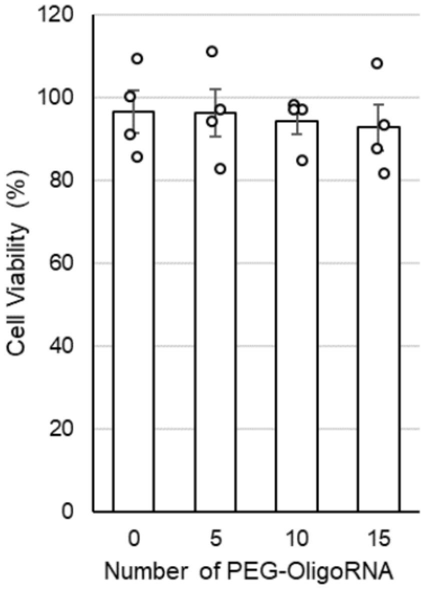

Figure S4. Cell viability of HuH-7 cells treated by each mRNA sample. HuH-7 was seeded on 96-well plate at a density of 10,000 cells/well in DMEM containing 10% FBS and 1% penicillin/ streptomycin in a humidified atmosphere with 5% CO_2 at 37 °C. After 24 h incubation, the culture medium was replaced with 100 μL of Opti-MEM, followed by adding each mRNA sample containing 250 ng of mRNA. After 4 h, culture medium was replaced with the 100 μL of fresh DMEM, and cells were incubated for additional 20 h. The cell viability was evaluated using CCK-8 assay kit (Dojindo laboratories, Kumamoto, Japan) by measuring absorbance at 450 nm. Data are shown as average mean ± SEM. Each dot refers to an individual measurement ($n = 4$).

Table S1. Sequences of PEG-OligoRNA.

	Sequences
PEG-OligoRNA #1	5′-(PEG)-AAAAACAGAACUUUGACUC-3′
PEG-OligoRNA #2	5′-(PEG)-AACUCGGCCACAGCGAUGC-3′
PEG-OligoRNA #3	5′-(PEG)-AAUUGAAGUCUUCGUUGUU-3
PEG-OligoRNA #4	5′-(PEG)-AAGGGCAACUUCCCGCGGU-3′
PEG-OligoRNA #5	5′-(PEG)-AAUCUUUGAGCACCUCCAG-3′
PEG-OligoRNA #6	5′-(PEG)-AAGCAGCCAGCUUUCCGGG-3′
PEG-OligoRNA #7	5′-(PEG)-AAGAUGAACUUCUUCAUCU-3′
PEG-OligoRNA #8	5′-(PEG)-AAACUCUUUGUCGCCUUCG-3′
PEG-OligoRNA #9	5′-(PEG)-AUAUCUCAGGAAUGUCGAC-3′
PEG-OligoRNA #10	5′-(PEG)-AUCACACAGAUCGACCUGU-3′
PEG-OligoRNA #11	5′-(PEG)-AAUUGAGGCAGCCAGUUGU-3′
PEG-OligoRNA #12	5′-(PEG)-AAAGAACACUGCACGUUGG-3′
PEG-OligoRNA #13	5′-(PEG)-UACUUGCUGGCAAAGGUCG-3′
PEG-OligoRNA #14	5′-(PEG)-AAUGAUCUUGUCCACCUGG-3′
PEG-OligoRNA #15	5′-(PEG)-AACUCUAGAUGCAUGCUCG-3′

Table S2. The number of hybridized PEG-OligoRNA with mRNA determined by GPC analysis.

Feeding amount of PEG-OligoRNA	5	10	15
The number of hybridized PEG-OligoRNA	5	10	14
